# Supplementary material for: Biodegradation of PET by the membrane-anchored PET esterase from the marine bacterium Rhodococcus pyridinivorans P23
Source: Commun Biol. 2023 Oct 27;6:1090. doi: 10.1038/s42003-023-05470-1 (PMC10611731; doi:10.1038/s42003-023-05470-1)
Supplement: Supplementary file 2 — SUPPLEMENTAL MATERIALS [file 42003_2023_5470_MOESM2_ESM.pdf]

SUPPLEMENTARY MATERIAL for

Biodegradation of PET by the membrane-anchored PET esterase from the marine bacterium *Rhodococcus pyridinivorans* P23

* **Corresponding authors:**

Dr. Wenbin Guo at: Key Laboratory of Marine Biogenetic Resources, Third Institute of Oceanography, Ministry of Natural Resources, Xiamen 361005, Fujian, China. E-mail addresses: guo5721@tio.org.cn; Tel: +86-592- 2195016.

Dr. Jingjing Duan at: College of Environment and Ecology, Xiamen University, Xiamen 361005, Fujian, China. E-mail addresses: duanjingjing@xmu.edu.cn; Tel: +86-592-2880204.

This supplementary materials includes supplementary tables, figures and amino acid sequence, nucleotide sequence and codon optimized nucleotide sequence of PET esterase (OQN32_06240).

Supplementary Table and Figure Legends

Supplementary Table 1 Potential PET hydrolase candidates in *R. pyridinovorans* P23

Supplementary Table 2 Putative *R. pyridinovorans* P23 proteins involved in TPA degradation

Supplementary Table3 The transcriptional profile of genes of *R. pyridinivorans* P23 involved in biodegradation of PET

Supplementary Table 4 Results of RNA-Seq read mapping onto *R. pyridinovorans* P23 genome

Supplementary Figure 1 Colony morphology of *Rhodococcus pyridinovorans* P23 cultured on 2216 Marine agar plate

Supplementary Figure 2 Neighbour-joining phylogenetic dendrogram, based on 16S rRNA gene sequences, for strain P23 and related species

Supplementary Figure 3 Growth curves for *R. pyridinovorans* P23 grown on either bis(hydroxyethyl) terephthalate (BHET) (a), mono(hydroxyethyl) terephthalate (MHET) (b), 2216 marine broth medium (c) or disodium terephthalate (TPA-Na_2) (d)

Supplementary Figure 4 BHET hydrolase activity tracking of *R. pyridinovorans* P23 cultured in 2216 marine broth medium

Supplementary Figure 5 Circular representation of *R. pyridinovorans* P23 genome

Supplementary Figure 6 Phylogenetic tree of 8 potential PET hydrolases with known PET hydrolytic enzymes *Is*-PETase, LCC and TfH.

Supplementary Figure 7 SDS-PAGE analysis of potential PET hydrolases expression in *E. coli* BL21 (DE3) and HPLC spectrum of BHET after enzymatic degradation with cell lysate of potential PET hydrolases

Supplementary Figure 8 Sequence analysis of PET esterase (OQN32_06240) of *R. pyridinivorans* P23

Supplementary Figure 9 The predicted three-dimensional structure of PET

esterase (OQN32_06240) protein

Supplementary Figure 10 Non-linear fit curves of PET esterase (OQN32_06240) towards BHET (A) and MHET (B) measured at pH 4.5, 30 ℃

Supplementary Figure 11 Gene organization of PET degradation related gene clusters from *R. pyridinovorans* P23

Supplementary Figure 12 Biodiversity of PET degrading bacteria in the environment possessing the same PET degradation pathway with *R. pyridinovorans* P23 using pfam search pattern

Supplementary Tables and Figures

Supplementary Table 1 Potential PET hydrolase candidates in *R. pyridinovorans* P23

gene	Location	gene _description	Super family	Number of TM domain	Predicted mature protein (a.a.)	Protein size of mature protein (kDa)	BHET degradation activity
OQN32_06240	chromosome	Esterase	Abhydrolase_3; Acetyl esterase/lipase; acetyl esterase	1	36-340	32.90	Yes
OQN32_01115	chromosome	Cutinase	Abhydrolase; Cutinase	1	32-319	31.23	No
OQN32_18580	chromosome	Alpha/beta-hydrolase	Abhydrolase_1; Pimeloyl-ACP methyl ester carboxylesterase	1	28-345	34.20	No
OQN32_15860	chromosome	Alpha/beta-hydrolase	Abhydrolase_9	4	155-434	29.43	No
OQN32_04705	chromosome	Alpha/beta-hydrolase	Abhydrolase_9	4	164-527	39.70	No
OQN32_03920	chromosome	Alpha/beta-hydrolase	Abhydrolase_9	5	218-591	41.67	No
OQN32_07550	chromosome	Alpha/beta-hydrolase	Abhydrolase_9	5	198-577	41.49	No
OQN32_07555	chromosome	Alpha/beta-hydrolase	Abhydrolase_9	5	199-573	40.00	No

Supplementary Table 2 Putative *R. pyridinovorans* P23 proteins involved in TPA degradation

ORF# (gene name) in *R. pyridinivorans* P23	Homolog(s) in *Ideonella sakaiensis* 201-F6 and *Rhodococcus jostii* RHA1				
	Gene	Identity (%)[a]	Gene_description	Source	Reference
OQN32_25270 (*pcaR*)	ISF6_0225	14.00%	pca regulon regulatory protein PcaR	*I. sakaiensis* 201-F6	1
OQN32_25275 (*pcaA*)	ISF6_0227	67.61%	Large subunit of the oxygenase component of TPA 1,2-dioxygenase (TPADO)	*I. sakaiensis* 201-F6	1
OQN32_25280 (*pcaC*)	ISF6_0228	55.19%	Small subunit of the oxygenase component of TPA 1,2-dioxygenase (TPADO)	*I. sakaiensis* 201-F6	1
OQN32_25285 (*pcaF*)	ISF6_0229	47.95%	1,2-dihydroxy-3,5-cyclohexadiene-1,4-dicarboxylate (DCD) dehydrogenase	*I. sakaiensis* 201-F6	1
OQN32_25290 (*pcaD*)	ISF6_0230	45.75%	Reductase component of TPA 1,2-dioxygenase (TPADO)	*I. sakaiensis* 201-F6	1
OQN32_25295 (*pcaK*)	ISF6_0226	33.91%	Probable terephthalate transporter, MFS superfamily	*I. sakaiensis* 201-F6	2
OQN32_09320 (*pcaG*)	ISF6_0626	58.52%	protocatechuate 3,4-dioxygenase, alpha chain	*I. sakaiensis* 201-F6	1
OQN32_09325 (*pcaH*)	ISF6_0627	61.18%	protocatechuate 3,4-dioxygenase, beta chain	*I. sakaiensis* 201-F6	1
OQN32_22690 (*scoB*)	-	62.10%	succinyl-CoA-3-ketoacid-CoA transferase subunit B	*I. sakaiensis* 201-F6 (GAP37024.1)	1
OQN32_22695 (*scoA*)	-	56.13%	succinyl-CoA-3-ketoacid-CoA transferase subunit A	*I. sakaiensis* 201-F6 (GAP37023.1)	1
OQN32_22700 (*pcaB*)	*pcaB*	57.02%	3-carboxy-cis, cis-muconate cycloisomerase	*Rhodococcus jostii* RHA (ABG93161)	3
OQN32_22705 (*pcaL*)	*pcaL*	61.94%	3-Oxoadipate enol-lactone hydrolase/ 4-carboxymuconolactone decarboxylase	*Rhodococcus jostii* RHA1 (ABG93162)	3
OQN32_22710 (*pcaR*)	*pcaR*	65.93	IclR family transcriptional regulator	*Rhodococcus jostii* RHA1 (ABG93163)	3
OQN32_22715 (*atoB*)	*pcaF*	80.20%	acetyl-CoA acetyltransferase	*Rhodococcus jostii* RHA1 (ABG93164)	3

[a] Identity with the corresponding *R. pyridinovorans* P23 protein was determined by their alignment using DNAMAN program.

Supplementary Table3 The transcriptional profile of genes of *R. pyridinivorans* P23 involved in biodegradation of PET

Gene_id	Gene_name	Gene_description	RPKM[a] (2216E)	RPKM (PET)	RPKM (TPA)	$Log_2(RPKM_{PET}/RPKM_{2216E})$ [b]	$Log_2(RPKM_{TPA}/RPKM_{2216E})$
		PET degradation					
OQN32_06240	-	esterase	75.61	34.17	70.72	-1.15	-0.10
		TPA degradation					
OQN32_25270	*pcaR*	pca regulon regulatory protein PcaR	20.25	21.12	254.78	0.06	3.65
OQN32_25275	*pcaA*	Large subunit of the oxygenase component of TPA 1,2-dioxygenase (TPADO)	8.03	89.47	60806.07	3.48	12.89
OQN32_25280	*pcaC*	Small subunit of the oxygenase component of TPA 1,2-dioxygenase (TPADO)	8.69	61.13	48374.41	2.81	12.44
OQN32_25285	*pcaF*	1,2-dihydroxy-3,5-cyclohexadiene-1,4-dicarboxylate (DCD) dehydrogenase	7.04	31.06	28346.7	2.14	11.98
OQN32_25290	*pcaD*	Reductase component of TPA 1,2-dioxygenase (TPADO)	11.6	32.72	29770.8	1.50	11.33
OQN32_25295	*pcaK*	MFS transporter	3.94	15.53	16043.44	1.98	11.99
		PCA degradation					
OQN32_09320	*pcaG*	protocatechuate 3,4-dioxygenase, alpha chain	8.44	16.82	2817.18	0.99	8.38
OQN32_09325	*pcaH*	protocatechuate 3,4-dioxygenase, beta chain	9.53	35.11	3022.41	1.88	8.31
OQN32_22690	*scoB*	succinyl-CoA-3-ketoacid-CoA transferase	30.71	36.7	992.81	0.26	5.01
OQN32_22695	*scoA*	succinyl-CoA-3-ketoacid-CoA transferase	84.4	95.8	1356.03	0.18	4.01
OQN32_22700	*pcaB*	3-carboxy-cis, cis-muconate cycloisomerase	11.14	13.92	80.76	0.32	2.86
OQN32_22705	*pcaL*	3-oxoadipate enol-lactone hydrolase	19.67	10.66	291.65	-0.88	3.89
OQN32_22710	*pcaR*	IclR family transcriptional regulator	33.93	14.07	455.67	-1.27	3.75

OQN32_22715	*atoB*	acetyl-CoA acetyltransferase	21.65	16.5	431.4	-0.39	4.32

[a] Averaged RPKM values of n=2 biological replicates of genes from *R. pyridinivorans* P23 cultured in 2216 marine broth medium and mineral salt medium with PET and TPA as sole carbon and energy source, respectively. RPKM, reads per kb per million reads. [b] $Log_2(RPKM_{PET}/RPKM_{2216E})$, the logarithmic value of Fold Change of RPKM values under PET and 2216E cultivation conditions.

Supplementary Table 4 Results of RNA-Seq read mapping onto *R. pyridinovorans* P23 genome

Culture medium	Biological replicate #	Raw reads	Raw Bases (bp)	Clean Reads	Clean Bases (bp)	Uniq Mapped Reads (bp) (Ratio(%))
PET film	1	53,131,308	8,022,827,508	52,313,712	6,168,633,222	36,868,905 (70.48%)
	2	48,086,358	7,261,040,058	47,180,464	5,657,426,189	27,554,953 (58.4%)
Disodium terephthalate ($TPA-Na_2$)	1	50,738,140	7,661,459,140	49,875,730	6,128,877,174	34,189,433 (68.55%)
	2	49,914,608	7,537,105,808	49,092,838	5,942,975,926	33,002,160 (67.22%)
2216E	1	31,011,330	4,682,710,830	30,498,234	4,235,556,482	29,386,103 (96.35%)
	2	29,206,832	4,410,231,632	28,677,856	4,051,191,962	27,733,459 (96.71%)

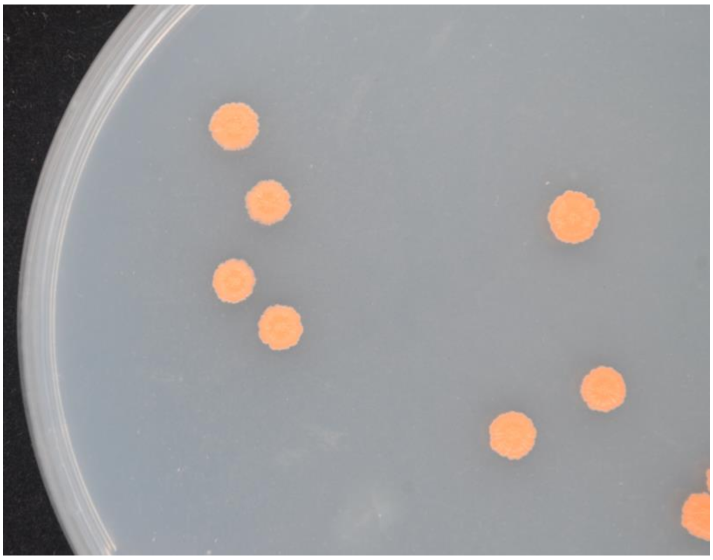

Supplementary Figure 1 Colony morphology of *Rhodococcus pyridinovorans* P23 cultured on 2216 marine agar plate

Rhodococcus pyridinovorans P23 was cultivated on 2216 marine agar medium in a 9 cm petri dish for 5- 10 days. The colonies were red with irregular edges, 3- 5 mm diameter.

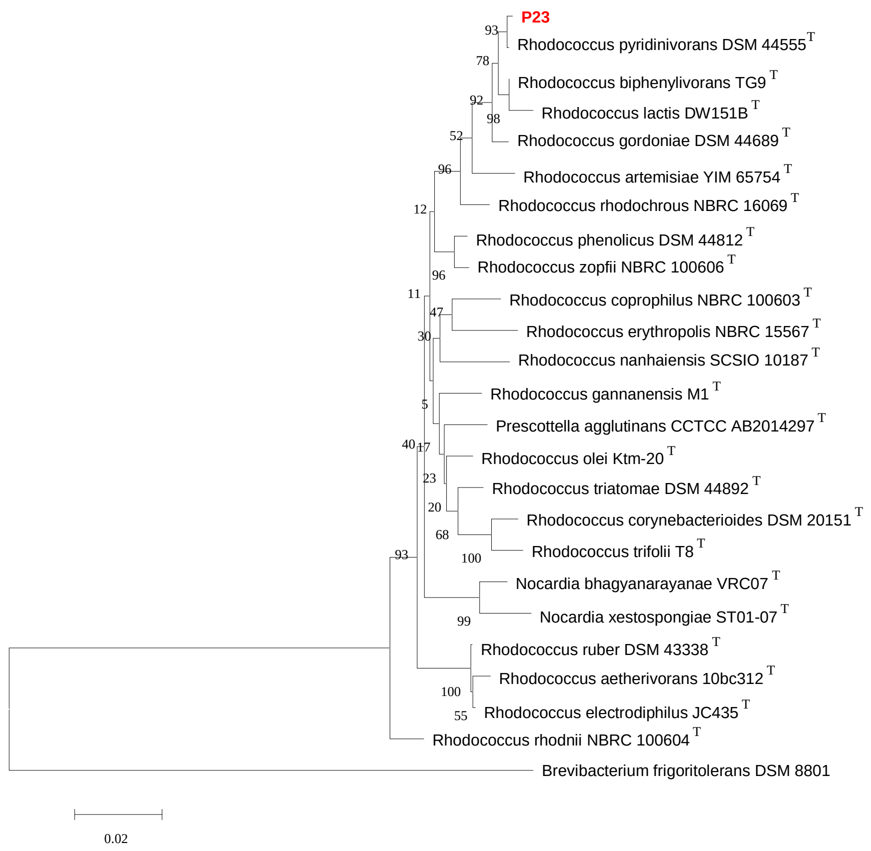

Supplementary Figure 2 Neighbour-joining phylogenetic dendrogram, based on 16S rRNA gene sequences, for strain P23 and related species

Analysis of the 16S rRNA gene sequence of strain P23 in EzbioCloud database (https://www.ezbiocloud.net/) showed that it belonged to the *Rhodococcus* genus. A neighbour-joining phylogenetic tree was constructed using 16S rRNA gene sequences of strain P23 and type strains of *Rhodococcus* genus. Bar, 2% sequence divergence. Out group, *Brevibacterium frigoritolerans* DSM 8801. Type strains are denoted via ^{T}.

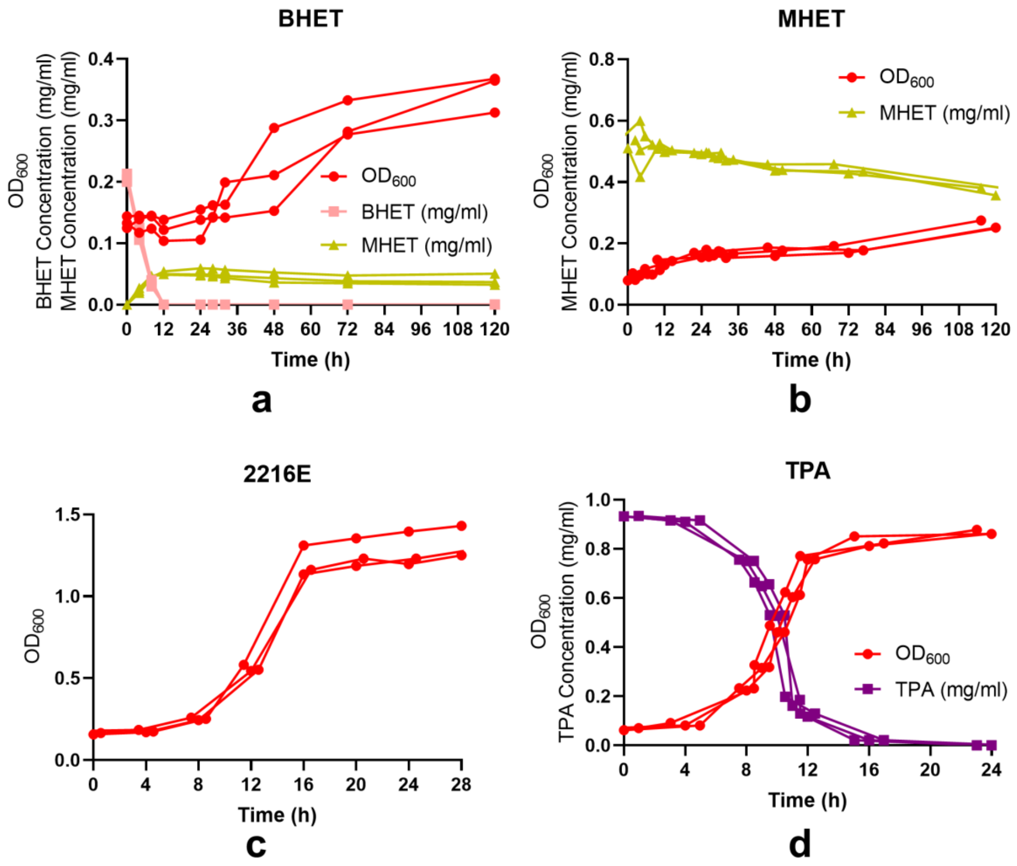

Supplementary Figure 3 Growth curves for *R. pyridinovorans* P23 grown on either bis(hydroxyethyl) terephthalate (BHET) (a), mono(hydroxyethyl) terephthalate (MHET) (b), 2216 marine broth medium (c) or disodium terephthalate (TPA-Na_2) (d)

R. pyridinovorans P23 was cultured in 150 ml 2216 marine broth medium or 150 ml of mineral salt medium with BHET (0.2 mg/ml), MHET (0.5 mg/ml), or TPA-Na_2 (1.0 g/l), and the optical density at 600 nm of the fluid was monitored. n= 3 biologically independent experiments were performed.

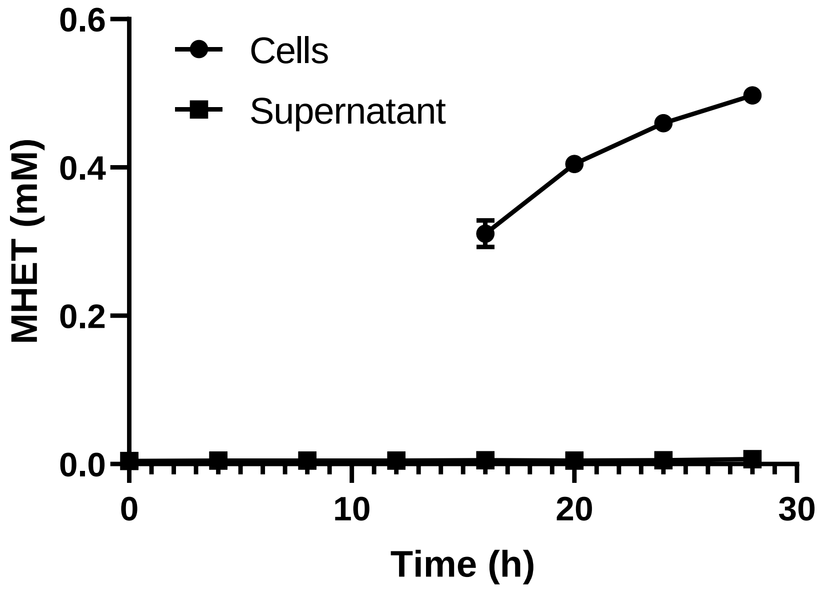

Supplementary Figure 4 BHET hydrolase activity tracking of *R. pyridinovorans* P23 cultured in 2216 marine broth medium

After cultivation of *R. pyridinovorans* P23 in 2216 marine broth medium, cells were separated from supernatant by centrifugation. Then cells and supernatant were tested for their BHET hydrolase activity, respectively. Result showed that the MHET accumulation increased from 0.31 mM to 0.50 mM in 16 to 28 h cultured cells due to the increase in the number of cells sampled, and no MHET production was detected in the supernatant sample. Obviously, the BHET hydrolase (PET hydrolase) locates on the cell membrane of *R. pyridinovorans* P23 exhibited definite BHET hydrolase activity while the supernatant not.

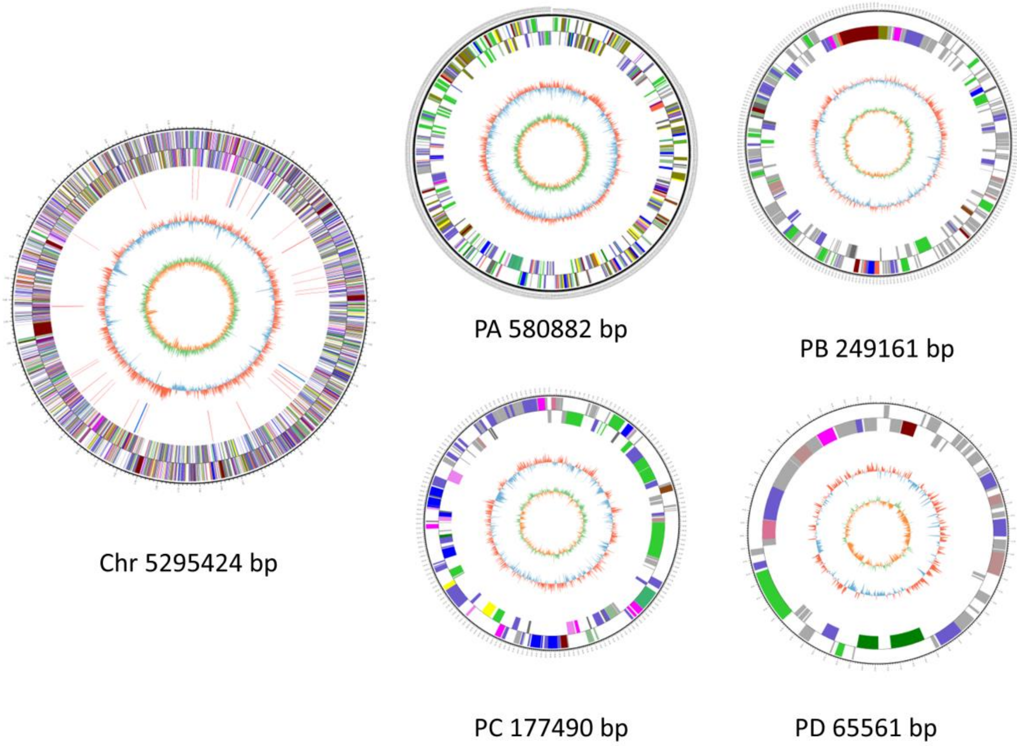

Supplementary Figure 5 Circular representation of *R. pyridinovorans* P23 genome

R. pyridinovorans P23 contains a circle chromosome of 5,295,424 bp with 68.20% GC content and 4 plasmids of 580,882 bp (plasmid A, 64.00% GC content), 249,161 bp (plasmid B, 63.37% GC content), 177,490 bp (plasmid C, 65.96% GC content), and 65,561 bp (plasmid D, 64.78% GC content). From the outside to the center: label of genome size, CDSs on the forward strand (colored by COG categories), CDSs on the reverse strand (colored by COG categories), rRNA and tRNA genes, G+ C content (peaks in red/blue indicate values higher or lower than average G+ C content, respectively), GC Skew. CDSs are depicted in different colors according to COG categories.

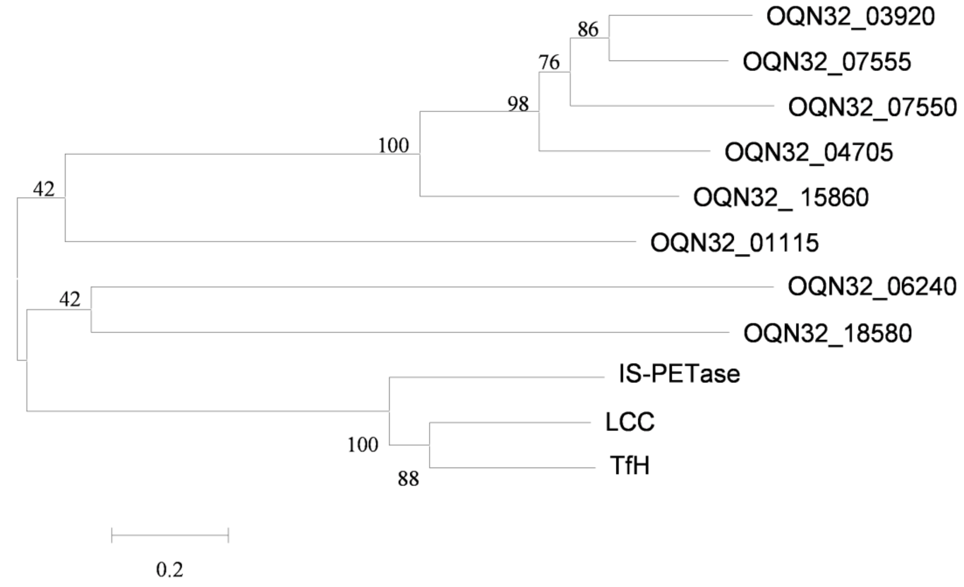

Supplementary Figure 6 Phylogenetic tree of 8 potential PET hydrolases with known PET hydrolytic enzymes *Is*-PETase, LCC and TfH.

These 8 potential PET hydrolases were compared to known PET degrading enzymes *Is*-PETase, LCC and TfH with a phylogenetic tree constructed using Mega 6.0. The GenBank accession numbers for *Is*-PETase,TfH and LCC are GAP38373.1, WP_011291330, and AEV21261 respectively. Bootstrap values are shown at the branch points. Scale bar, 0.2 amino acid substitutions per single site.

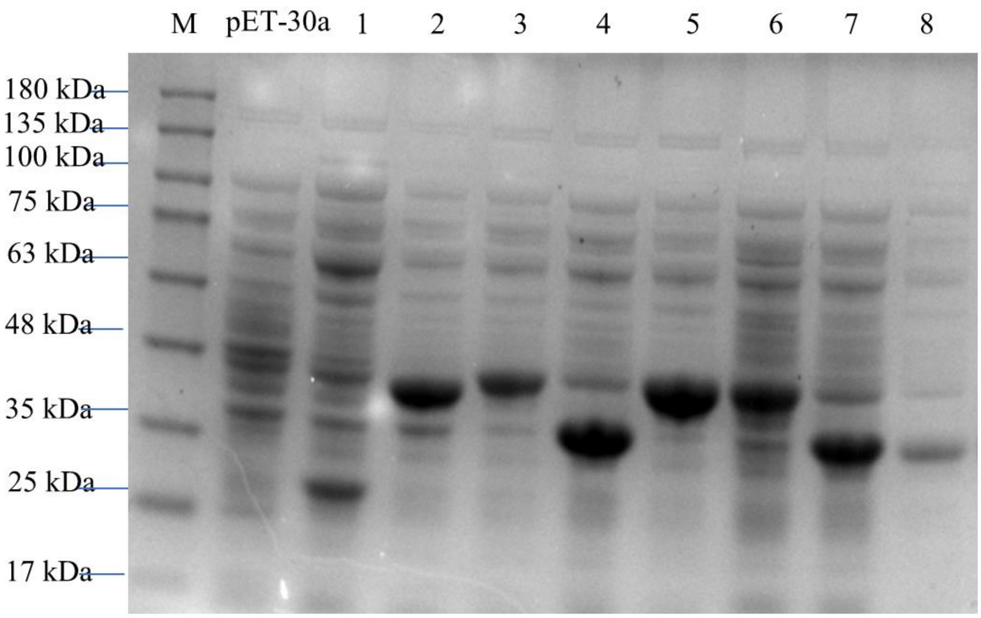

a

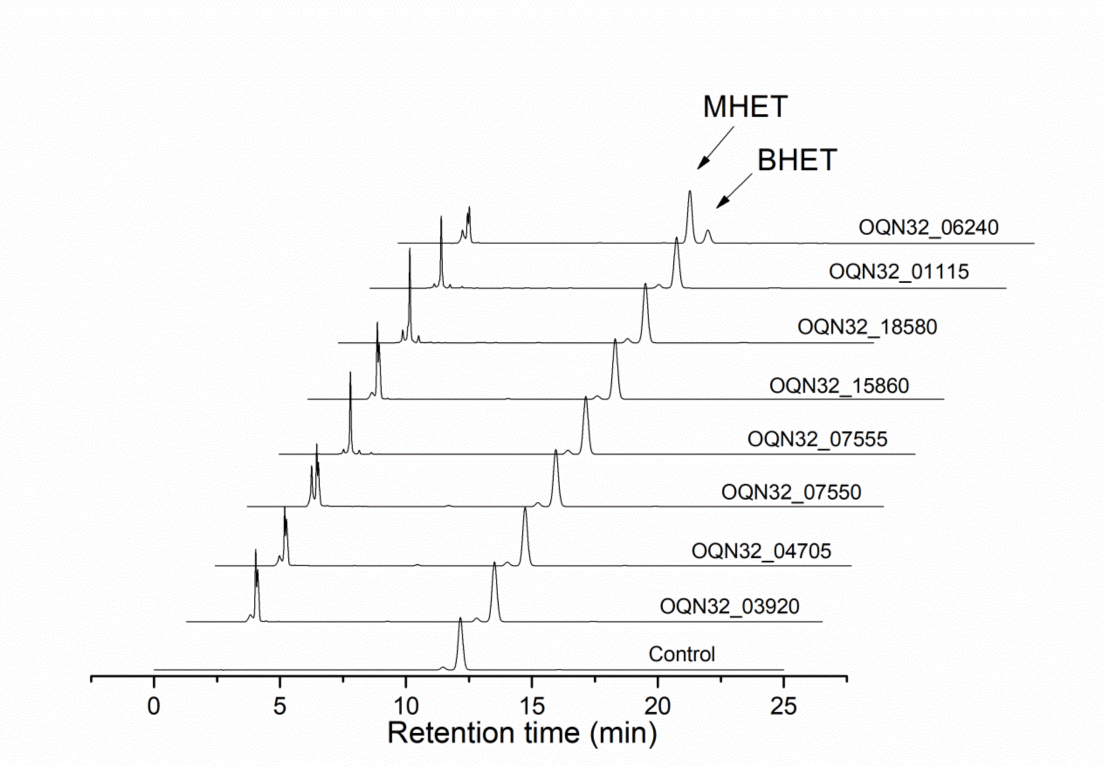

b

Supplementary Figure 7 SDS-PAGE analysis of potential PET hydrolases expression in *E. coli* BL21 (DE3) and HPLC spectrum of BHET after enzymatic

degradation with cell lysate of potential PET hydrolases

After the transmembrane domains were removed, the genes for potential PET hydrolases (see Table S1) were commercially synthesized with codon optimization for expression in *Escherichia coli* BL21 (DE3) (Sangon, Shanghai, China). The potential PET hydrolases were expressed in *E. coli* BL21 (DE3) by 0.5 mM IPTG induction at 20 ℃. The *E. coli* cells were harvested by centrifugation (4,000 × g, 10 min, 4 ℃), resuspended in lysis buffer (50 mM Tris-HCl, 300 mM NaCl, 0.1% Triton X-100, pH 8.0) and disrupted by sonication on ice. After centrifugation (12,000 × g, 15 min, 4 ℃), insoluble debris was removed, and the supernatant containing the soluble protein was subjected to SDS-PAGE analysis and BHET hydrolysis. Results showing that the eight proteins were all successfully synthesized in soluble form in the supernatant of *E. coli* lysates (a), and only the cell lysate of OQN32_06340 could degrade BHET into MHET (b). a, M, protein marker (Solarbio Life Sciences, Beijing, China), pET-30a, cell lysate of *E. coli* BL21 (DE3) harboring plasmid pET-30a as negative control, lane 1- 8, cell lysate of *E. coli* BL21 (DE3) expressing corresponding protein of OQN32_15860, OQN32_04705, OQN32_03920, OQN32_18580, OQN32_07550, OQN32_07555, OQN32_06240, and OQN32_01115, respectively.

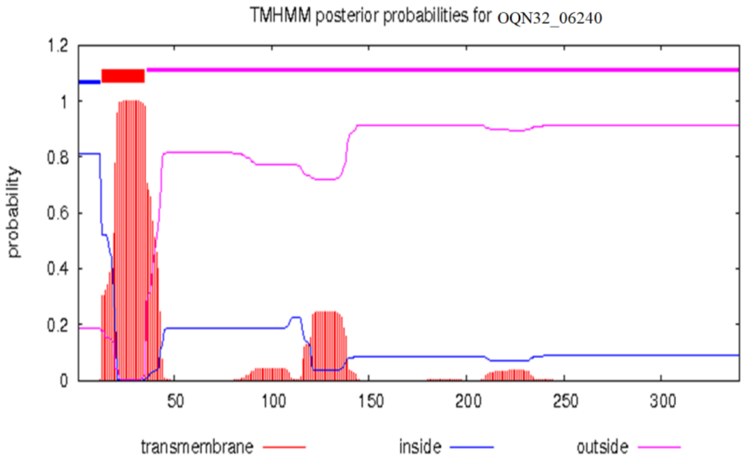

a

1EVQ	-----------MPLDPVIQQVLDQLNRMPAPDYKHLSAQQ	29
OQN32_06240	MAEDVTESRPRRPIGPRVVLVLAFVVVVTAAAFVLTPVPG	40
	* * ** *	
1EVQ	FRSQQSLFPPVKKEPVAEVR------EFDMDLPGR----T	59
OQN32_06240	SLVVRKVFERDAREQTEKLAVDAPETDFVADLHYREDDPD	80
	* * * ** *	
1EVQ	LKVRMYRPEGVEPPYPALVYYHGGGWVVGDLETHDPVCRV	99
OQN32_06240	AYLDVYTPPGTTEALPTIVWTHGGAWLSGNRTNYAGYYRR	120
	* * * * * *** * * *	
1EVQ	LAKDGRAVVFSVDYRLAPEHKFPAAVEDAYDALQWIAERA	139
OQN32_06240	LAAAG-FTVVSVGYSLAPGHRYPTPVRQLVDAQRYLLEHA	159
	** * * ** * *** * * * ** * *	
	GXSXG	
1EVQ	ADFHLDPARIAVGGDSAGGNLAAVTSILAKERGGPALAFQ	179
OQN32_06240	DELHIDTERIVLAGDSAGAQLSAQIAAAVTDPDYAALLGV	199
	* * ** ***** * * **	
1EVQ	LLIYPSTGYDPAHPPASIEENAEGYLLTGGMMLWFRDQYL	219
OQN32_06240	DPAFTPENVRGVVLNCGIYD-VSAIGGSGGLIGWGVEQAM	238
	* ** * *	
1EVQ	NSLEELTHPWFS------PVLYPDLSGLPPAYIATAQYDP	253
OQN32_06240	WAYTGAREFATSDAAGQMSVLNSVTENFPATYISGGNADP	278
	* ** * ** **	
1EVQ	LRDVG-KLYAEALNKAGVKVEIENFED----LIHGFAQFY	288
OQN32_06240	LTATQSERLAQRLTGLGVQVDALFYPDDHTPELAHEYQFD	318
	* * * ** * * **	
1EVQ	SLSPGATKALVRIAEKLRDALA	309
OQN32_06240	LSTPDARAALERTIDFVRKVTT	339
	* * ** * *	

b

Supplementary Figure 8 Sequence analysis of PET esterase (OQN32_06240) of *R. pyridinivorans* P23

a, Transmembrane domain of PET esterase (OQN32_06240) was predicted using the TMHMM server V2.0 showing a 13- 35 amino acid residues of transmembrane domain. b, Amino acid sequence alignment of PET esterase (OQN32_06240) and esterase 2 (EST2) from *Alicyclobacillus acidocaldarius*. The amino acid residues ($S_{155}D_{252}H_{282}$ in 1EVQ and $S_{175}D_{277}H_{313}$ in OQN32_06240) forming a catalytic triad (shaded in yellow) were identified. The GXSXG motif conserved in α/β-fold hydrolases is underlined. Conserved residues are indicated with *. The transmembrane motif predicted in the N terminal of PET Esterase (OQN32_06240) is shaded in green.

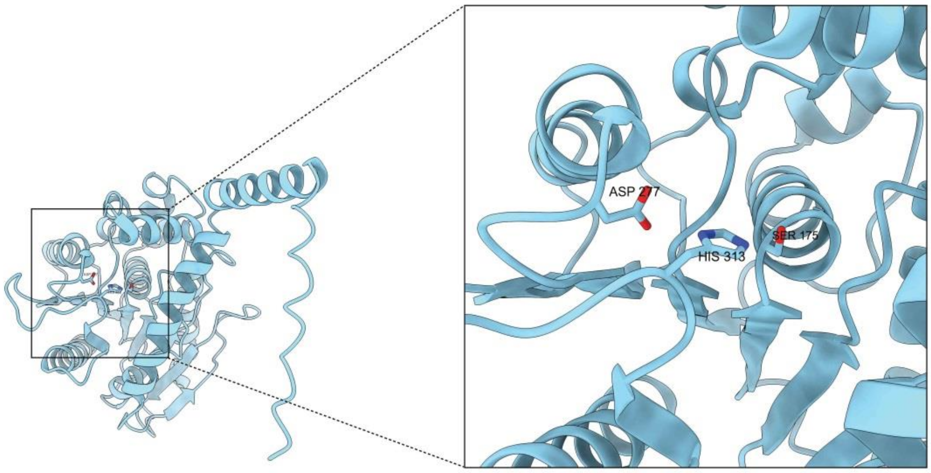

Supplementary Figure 9 The predicted three-dimensional structure of PET esterase (OQN32_06240) protein

The three-dimensional structure of PET esterase (OQN32_06240) was predicted with AlphaFold2, and the amino acid residues $S_{175}D_{277}H_{313}$ forming a catalytic triad were identified.

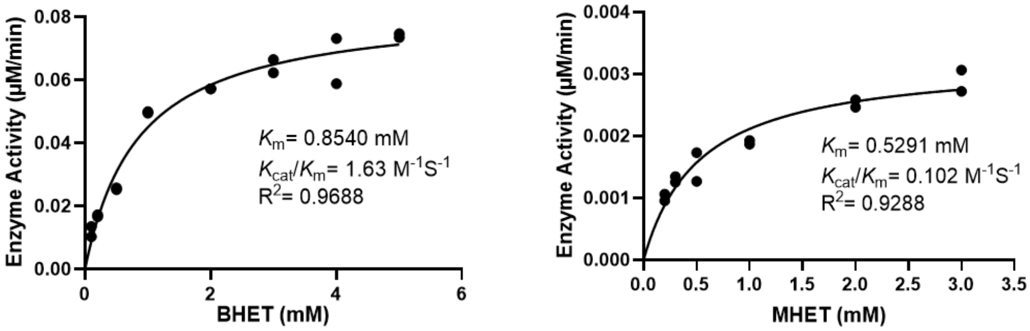

a b

Supplementary Figure 10 Non-linear fit curves of PET esterase (OQN32_06240) towards BHET (a) and MHET (b) measured at pH 4.5, 30 ℃

Two replicates were performed in each substrate concentration.

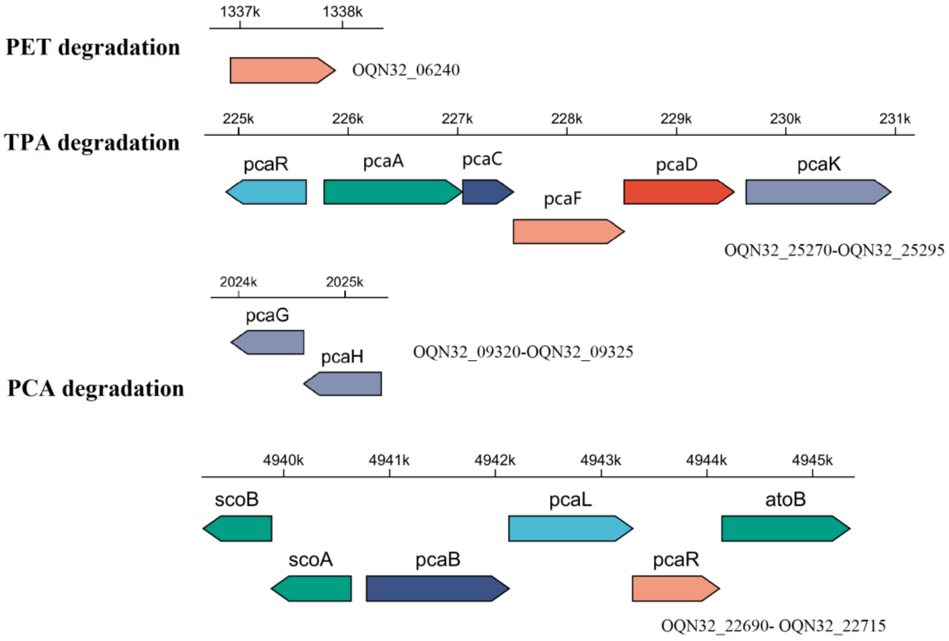

Supplementary Figure 11 Gene organization of PET degradation related gene clusters from *R. pyridinovorans* P23

PET degradation related gene clusters of *R. pyridinovorans* P23 includes PET degradation esterase (OQN_06240), TPA degradation operon in pA plasmid, and PCA degradation operons in chromosome. The scale of gene size was also present to indicate the positions of genes in genome and pA plasmid.

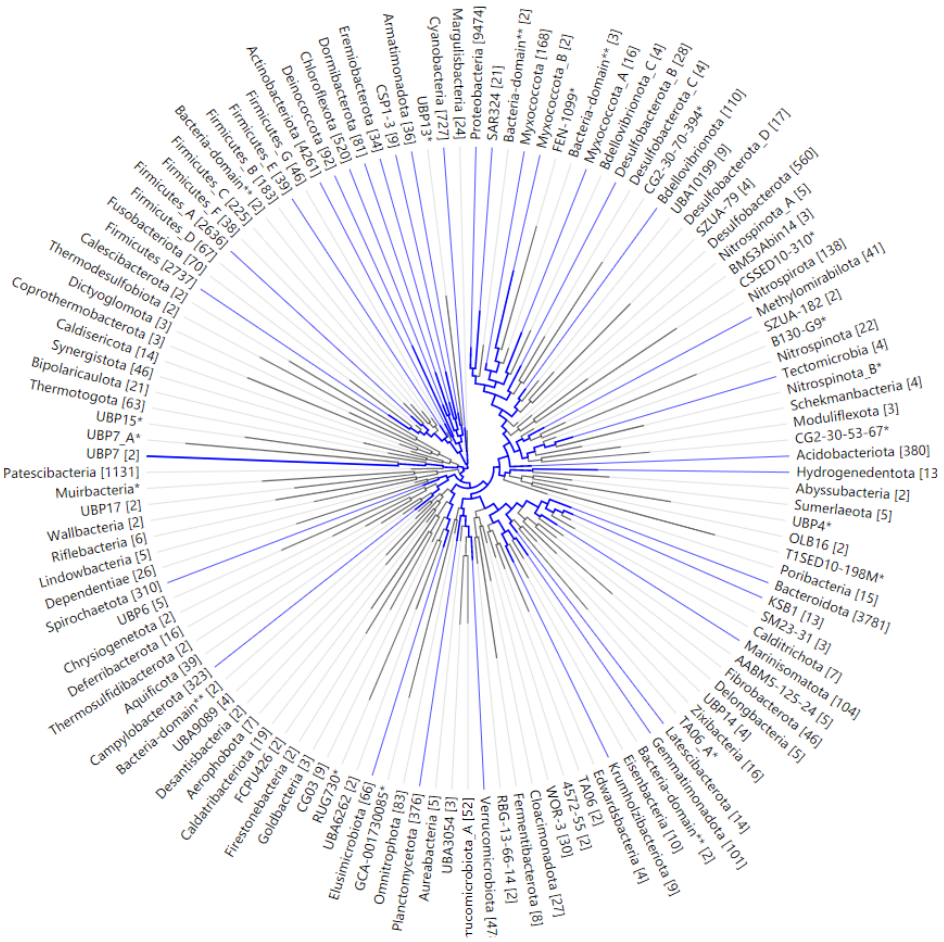
Proteobacteria [9474]
SAR324 [21]
Bacteria-domain** [2]
Myxococcota [168]
Myxococcota_B [2]
FEN-1099*
Bacteria-domain** [3]
Myxococcota_A [16]
Bdellovibrionota_C [4]
Desulfobacterota_B [28]
Desulfobacterota_C [4]
CG2-30-70-394*
Bdellovibrionota [110]
UBA10199 [9]
Desulfobacterota_D [17]
SZUA-79 [4]
Desulfobacterota [560]
Nitrospinota_A [5]
BMS3Abin14 [3]
CSSED10-310*
Nitrospirota [138]
Methylomirabilota [41]
SZUA-182 [2]
B130-G9*
Nitrospinota [22]
Tectomicrobia [4]
Nitrospinota_B*
Schekmanbacteria [4]
Moduliflexota [3]
CG2-30-53-67*
Acidobacteriota [380]
Hydrogenedentota [13
Abyssubacteria [2]
Sumerlaeota [5]
UBP4*
OLB16 [2]
T1SED10-198M*
Poribacteria [15]
Bacteroidota [3781]
KSB1 [13]
SM23-31 [3]
Calditrichota [7]
Marinisomatota [104]
AABM5-125-24 [5]
Fibrobacterota [46]
Delongbacteria [5]
Zixibacteria [16]
UBP14 [4]
TA06_A*
Latescibacterota [14]
Gemmatimonadota [101]
Bacteria-domain** [2]
Eisenbacteria [10]
Krumholzibacteriota [9]
Edwardsbacteria [4]
TA06 [2]
4572-55 [2]
WOR-3 [30]
Cloacimonadota [27]
Fermentibacterota [8]
RBG-13-66-14 [2]
Verrucomicrobiota [47
rucomicrobiota_A [52]
UBA3054 [3]
Aureabacteria [5]
Planctomycetota [376]
Omnitrophota [83]
GCA-001730085*
Elusimicrobiota [66]
UBA6262 [2]
RUG730*
CG03 [9]
Goldbacteria [3]
Firestonebacteria [2]
FCPU426 [2]
Caldatribacteriota [19]
Aerophobota [7]
Desantisbacteria [2]
UBA9089 [4]
Bacteria-domain** [2]
Campylobacterota [323]
Aquificota [39]
Thermosulfidibacterota [2]
Deferribacterota [16]
Chrysiogenetota [2]
UBP6 [5]
Spirochaetota [310]
Dependentiae [26]
Lindowbacteria [5]
Riflebacteria [6]
Wallbacteria [2]
UBP17 [2]
Muirbacteria*
Patescibacteria [1131]
UBP7 [2]
UBP7_A*
UBP15*
Thermotogota [63]
Bipolaricaulota [21]
Synergistota [46]
Caldisericota [14]
Coprothermobacterota [3]
Dictyoglomota [3]
Thermodesulfobiota [2]
Calescibacterota [2]
Firmicutes [2737]
Fusobacteriota [70]
Firmicutes_D [67]
Firmicutes_A [2636]
Firmicutes_F [38]
Firmicutes_C [225]
Bacteria-domain** [2]
Firmicutes_B [183]
Firmicutes_E [39]
Firmicutes_G [46]
Actinobacteriota [4261]
Deinococcota [92]
Chloroflexota [520]
Dormibacterota [81]
Eremiobacterota [34]
CSP1-3 [9]
Armatimonadota [36]
UBP13*
Cyanobacteria [727]
Margulisbacteria [24]

a

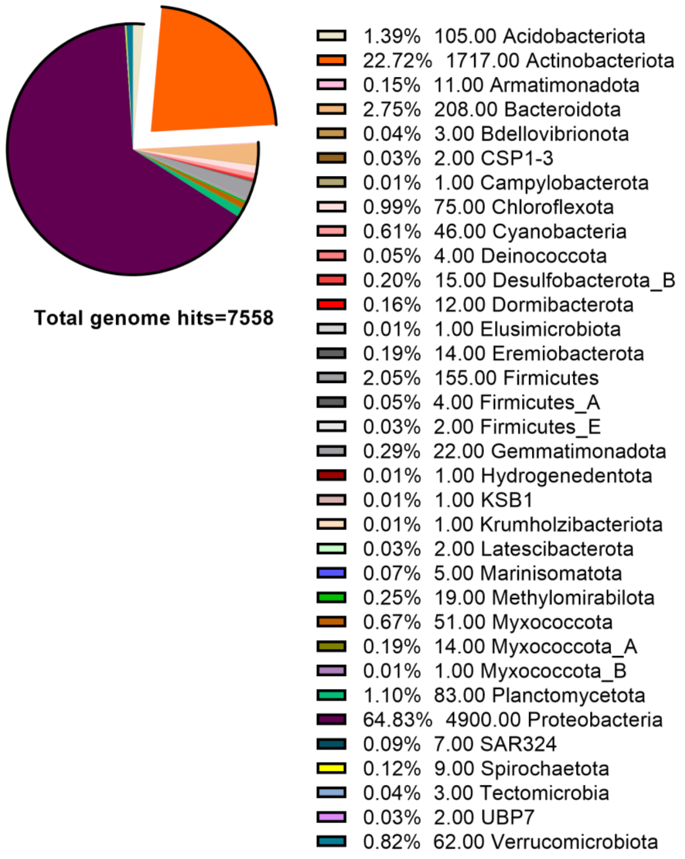

b

Supplementary Figure 12 Biodiversity of PET degrading bacteria in the environment possessing the same PET degradation pathway with *R. pyridinovorans* P23 using pfam search pattern

Genome hits with the coexistence of PET esterase (PF07859) and large subunit of the oxygenase component of TPA 1,2-dioxygenase (TPADO) (PF00848) in the phylum level showing *Proteobacteria* and *Actinobacteriota* as the main compositions. a, Blue lines in indicate genome hits of bacteria in the phylum level. Numbers in square brackets shows the totally genomes in the database. b, Bacteria composition of genome hits in the phylum level showing *Proteobacteria* and *Actinobacteriota* as the main compositions.

Amino acid sequences of PET esterase (OQN32_06240):

12- 35 a.a. shaded in green is the transmembrane domain. The first 35 a.a. underlined would be removed in heterologous expression in *Escherichia coli* BL21(DE3).

> OQN32_06240
MAEDVTESRPRRPIGPRVVLVLAFVVVVTAAAFVLTPVPGSLVVRKVFERDAR
EQTEKLAVDAPETDFVADLHYREDDPDAYLDVYTPPGTTEALPTIVWTHGGA
WLSGNRTNYAGYYRRLAAAGFTVVSVGYSLAPGHRYPTPVRQLVDAQRYLL
EHADELHIDTERIVLAGDSAGAQLSAQIAAAVTDPDYAALLGVDPAFTPENVR
GVVLNCGIYDVSAIGGSGGLIGWGVEQAMWAYTGAREFATSDAAGQMSVLN
SVTENFPATYISGGNADPLTATQSERLAQRLTGLGVQVDALFYPDDHTPELAH
EYQFDLSTPDARAALERTIDFVRKVTT

Nucleotide sequence of coding gene of PET esterase (OQN32_06240):

The initiation and termination codons are shaded in red. The first 35 codons underlined would be removed.

> OQN32_06240
TTGGCCGAAGACGTCACCGAGAGCAGGCCTCGTCGCCCGATCGGACCCCG
GGTCGTGCTCGTTCTGGCATTCGTCGTGGTGGTCACGGCCGCCGCATTCGT
CCTCACCCCCGTGCCGGGGTCACTGGTGGTGCGCAAGGTGTTCGAACGCG
ACGCGCGGGAGCAGACCGAGAAGCTCGCGGTGGACGCTCCGGAGACGGA
CTTCGTCGCCGACCTCCACTACCGGGAGGACGACCCGGACGCCTACCTCG
ACGTGTACACACCGCCAGGGACCACCGAGGCGCTGCCGACGATCGTGTGG
ACGCACGGCGGCGCATGGTTGTCGGGTAATCGCACCAACTACGCGGGTTAC
TACAGGCGTCTCGCGGCCGCCGGATTCACTGTGGTGTCGGTCGGCTACTCG
CTCGCACCGGGCCATCGCTATCCGACCCCGGTGCGGCAACTCGTCGATGCA
CAGCGCTACCTGCTCGAGCACGCCGACGAACTGCACATCGACACCGAGCG
GATCGTGCTCGCCGGTGACTCGGCCGGGGCGCAACTGTCCGCGCAGATCG
CCGCGGCGGTCACCGACCCCGACTACGCCGCGCTCCTCGGTGTCGACCCG
GCGTTCACACCGGAGAACGTGCGCGGTGTCGTGCTCAACTGCGGCATCTA
CGACGTGTCCGCGATCGGTGGGAGCGGCGGGCTGATCGGATGGGGCGTCG
AGCAGGCGATGTGGGCCTACACCGGCGCCCGGGAATTCGCCACTTCGGAT
GCGGCGGGCCAGATGTCGGTGTTGAACTCGGTCACCGAGAACTTCCCGGC
CACCTACATTTCCGGTGGCAACGCCGATCCGCTCACCGCCACCCAGTCCGA
GAGGCTCGCGCAGCGACTCACCGGACTGGGTGTGCAGGTGGATGCGTTGT
TCTATCCGGACGATCACACACCGGAACTGGCACACGAGTACCAGTTCGATC
TGTCCACGCCGGATGCGCGTGCCGCGCTCGAGCGGACGATCGATTTCGTCC
GCAAGGTCACGACGTAG

Codon optimized nucleotide sequence of coding gene of PET esterase

(OQN32_06240):

The *Nde*I (5′ end) and *Xho*I (3′ end) sites are shaded in yellow.

> OQN32_06240 (codon optimized)
CATATGACCCCAGTACCTGGTAGCCTGGTAGTGCGTAAGGTGTTCGAGCGT
GACGCACGTGAGCAAACCGAGAAACTGGCGGTGGACGCTCCTGAAACGG
ACTTCGTCGCAGACCTGCATTACCGCGAGGATGACCCAGACGCATACCTGG
ACGTGTACACGCCACCGGGTACTACTGAAGCGCTGCCGACTATCGTGTGGA
CTCACGGTGGTGCATGGCTGAGCGGTAACCGTACTAACTACGCGGGTTACT
ACCGCCGTCTGGCAGCTGCTGGTTTCACCGTCGTGAGCGTCGGTTACAGCC
TGGCACCGGGTCATCGTTACCCGACTCCGGTTCGTCAACTGGTTGATGCAC
AGCGTTACCTGCTGGAACACGCAGATGAACTGCACATCGACACCGAACGC
ATCGTCCTGGCAGGTGATTCCGCTGGTGCTCAGCTGTCCGCTCAGATCGCT
GCCGCCGTTACTGACCCGGATTACGCTGCTCTGCTGGGTGTGGATCCGGCT
TTCACTCCGGAAAACGTTCGCGGTGTTGTTCTGAACTGCGGCATCTATGAC
GTTTCCGCGATCGGCGGCTCCGGTGGCCTGATTGGCTGGGGCGTTGAACAG
GCTATGTGGGCATATACCGGCGCGCGTGAATTCGCCACGTCTGATGCCGCG
GGCCAGATGTCTGTTCTGAATTCCGTAACCGAAAACTTCCCGGCGACCTATA
TTTCTGGCGGCAACGCGGACCCGCTGACCGCGACCCAGTCTGAACGTCTG
GCGCAGCGTCTGACCGGCCTGGGCGTTCAGGTTGATGCCCTGTTTTATCCG
GATGATCACACCCCGGAACTGGCCCACGAATATCAGTTTGATCTGTCTACG
CCGGACGCGCGTGCGGCGCTGGAACGCACCATTGACTTTGTACGCAAAGT
AACCACCCTCGAG

References:

1. Yoshida, S. et al. A bacterium that degrades and assimilates poly(ethylene terephthalate). *Science* **351**, 1196-1199 (2016).
2. Nichols, N. N. & Harwood, C. S. PcaK, a high-affinity permease for the aromatic compounds 4-hydroxybenzoate and protocatechuate from Pseudomonas putida. *J Bacteriol* **179**, 5056-5061 (1997).
3. Hara, H., Eltis, L. D., Davies, J. E. & Mohn, W. W. Transcriptomic analysis reveals a bifurcated terephthalate degradation pathway in Rhodococcus sp. strain RHA1. *J Bacteriol* **189**, 1641-1647 (2007).
