# Supplementary material for: Biodegradation of PET by the membrane-anchored PET esterase from the marine bacterium Rhodococcus pyridinivorans P23
Source: Commun Biol. 2023 Oct 27;6:1090. doi: 10.1038/s42003-023-05470-1 (PMC10611731; doi:10.1038/s42003-023-05470-1)
Supplement: Supplementary file 3 — Description of Additional Supplementary Files [file 42003_2023_5470_MOESM3_ESM.pdf]

Description of Additional Supplementary Files

File name: Supplementary Data 1
Description: Differentially transcribed genes identified using the MajorBio platform.

File name: Supplementary Data 2
Description: The source data underlying the graphs for Fig. 2a-2b, Fig. 4a- 4d, Fig. 5a- 5f and Fig. 7 in the paper.
