# Supplementary material for: Biodegradation of PET by the membrane-anchored PET esterase from the marine bacterium Rhodococcus pyridinivorans P23
Source: Commun Biol. 2023 Oct 27;6:1090. doi: 10.1038/s42003-023-05470-1 (PMC10611731; doi:10.1038/s42003-023-05470-1)
Supplement: Supplementary file 6 — Reporting Summary [file 42003_2023_5470_MOESM6_ESM.pdf]

nature portfolio

Corresponding author(s): Wenbin Guo; Jingjing Duan

Last updated by author(s): Oct 11, 2023

Reporting Summary

Nature Portfolio wishes to improve the reproducibility of the work that we publish. This form provides structure for consistency and transparency in reporting. For further information on Nature Portfolio policies, see our Editorial Policies and the Editorial Policy Checklist.

Statistics

For all statistical analyses, confirm that the following items are present in the figure legend, table legend, main text, or Methods section.

n/a	Confirmed	
☐	☒	The exact sample size (*n*) for each experimental group/condition, given as a discrete number and unit of measurement
☐	☒	A statement on whether measurements were taken from distinct samples or whether the same sample was measured repeatedly
☒	☐	The statistical test(s) used AND whether they are one- or two-sided<br>*Only common tests should be described solely by name; describe more complex techniques in the Methods section.*
☒	☐	A description of all covariates tested
☒	☐	A description of any assumptions or corrections, such as tests of normality and adjustment for multiple comparisons
☒	☐	A full description of the statistical parameters including central tendency (e.g. means) or other basic estimates (e.g. regression coefficient) AND variation (e.g. standard deviation) or associated estimates of uncertainty (e.g. confidence intervals)
☒	☐	For null hypothesis testing, the test statistic (e.g. *F*, *t*, *r*) with confidence intervals, effect sizes, degrees of freedom and *P* value noted<br>*Give P values as exact values whenever suitable.*
☒	☐	For Bayesian analysis, information on the choice of priors and Markov chain Monte Carlo settings
☒	☐	For hierarchical and complex designs, identification of the appropriate level for tests and full reporting of outcomes
☒	☐	Estimates of effect sizes (e.g. Cohen's *d*, Pearson's *r*), indicating how they were calculated

Our web collection on statistics for biologists contains articles on many of the points above.

Software and code

Policy information about availability of computer code

Data collection	Agilent 1260 infinity system was used to collect the HPLC data. The online Annotree server (http://annotree.uwaterloo.ca/app/) was utilized to search the coexistence of PET esterase (PF07859, K01066) and large subunit of the oxygenase component of TPADO (PF00848, K16319) homologs in bacteria. The three-dimensional structure of PET esterase (OQN32_06240) was predicted with AlphaFold2. The genome sequence and differentially transcribed genes analysis were performed using the MajorBio platform (https://cloud.majorbio.com/). The bacterial growth images on PET were collected from environmental scanning electron microscope (SEM, FEI Quanta450, USA).
Data analysis	GraphPad Prism 8 was used to analyze the data involving in enzyme characterization, bacterial growth and PET weight loss. Origin 9.0 was used to build the HPLC chromatogram which was collected from Agilent 1260 infinity system. The phylogenetic tree was constructed using the Mega 6.0 program using Neighbour-joining method.

For manuscripts utilizing custom algorithms or software that are central to the research but not yet described in published literature, software must be made available to editors and reviewers. We strongly encourage code deposition in a community repository (e.g. GitHub). See the Nature Portfolio guidelines for submitting code & software for further information.

Data

Policy information about availability of data

All manuscripts must include a data availability statement. This statement should provide the following information, where applicable:

- Accession codes, unique identifiers, or web links for publicly available datasets
- A description of any restrictions on data availability
- For clinical datasets or third party data, please ensure that the statement adheres to our policy

The complete genome sequence of R. pyridinovorans P23 was deposited in the GenBank database under the accession number CP113798-CP113802. The RNA-Seq reads have been deposited in GenBank with accession number SRR23047356- SRR23047361.

Research involving human participants, their data, or biological material

Policy information about studies with human participants or human data. See also policy information about sex, gender (identity/presentation), and sexual orientation and race, ethnicity and racism.

Reporting on sex and gender	No human participant involved in our study.
Reporting on race, ethnicity, or other socially relevant groupings	No human participant involved in our study.
Population characteristics	No human participant involved in our study.
Recruitment	No human participant involved in our study.
Ethics oversight	No human participant involved in our study.

Note that full information on the approval of the study protocol must also be provided in the manuscript.

Field-specific reporting

Please select the one below that is the best fit for your research. If you are not sure, read the appropriate sections before making your selection.

☒ Life sciences ☐ Behavioural & social sciences ☐ Ecological, evolutionary & environmental sciences

For a reference copy of the document with all sections, see nature.com/documents/nr-reporting-summary-flat.pdf

Life sciences study design

All studies must disclose on these points even when the disclosure is negative.

Sample size	For experiments involving in quantification of bacterial growth and PET weight loss, n=3 was chosen as the replicate number. For experiments involving in quantification of enzyme activities, n=3 was chosen as the replicate number. For the RNA-Seq samples, n=2 was chosen as the replicate number.
Data exclusions	Data were not excluded from analysis.
Replication	The purified PET esterase and whole cell biocatalysis presented the same characteristic towards PET, BHET and MHET under pH3.0- 8.0 conditions.
Randomization	Randomization was not required in our experiments.
Blinding	Blinding was not possible as experimental conditions were evident in our study with a certain enzyme from a certain strain.

Reporting for specific materials, systems and methods

We require information from authors about some types of materials, experimental systems and methods used in many studies. Here, indicate whether each material, system or method listed is relevant to your study. If you are not sure if a list item applies to your research, read the appropriate section before selecting a response.

Materials & experimental systems

n/a	Involved in the study
☒	☐ Antibodies
☒	☐ Eukaryotic cell lines
☒	☐ Palaeontology and archaeology
☒	☐ Animals and other organisms
☒	☐ Clinical data
☒	☐ Dual use research of concern
☒	☐ Plants

Methods

n/a	Involved in the study
☒	☐ ChIP-seq
☒	☐ Flow cytometry
☒	☐ MRI-based neuroimaging
